# Is there an Association between Dietary Micronutrients Intake and Bone Fractures among Malaysian Reproductive-Age Women? The PURE Malaysia Study

**DOI:** 10.5334/aogh.4445

**Published:** 2024-09-04

**Authors:** Zaleha Md Isa, Nur Atiqah Mohd Ahwan, Noor Hassim Ismail, Rosnah Ismail, Azmi Mohd Tamil, Mohd Hasni Jaafar, Nafiza Mat-Nasir, Nik Munirah Nik Mohd Nasir, Nurul Hafiza Ab Razak, Khairul Hazdi Yusof

**Affiliations:** 1 Department of Public Health Medicine, Faculty of Medicine, UKM Medical Centre, Universiti Kebangsaan Malaysia, Kuala Lumpur, Cheras, Malaysia; 2 Department of Primary Care Medicine, Faculty of Medicine, Universiti Teknologi MARA (UiTM), Sungai Buloh Campus, Selangor Branch, Jalan Hospital, Sungai Buloh, Selangor, Malaysia; Hospital al-Sultan Abdullah, Universiti Teknologi MARA, 42300 Bandar Puncak Alam, Selangor, Malaysia; 3 Risk Management Unit, Director Office, Hospital Canselor Tuanku Muhriz, UKM Medical Centre, Universiti Kebangsaan Malaysia, Kuala Lumpur, Cheras, Malaysia

**Keywords:** bone fractures, reproductive-age women, micronutrient, vitamin, cross-sectional

## Abstract

*Background:* Bone fractures represent a significant health issue and impose a considerable burden on healthcare systems globally. However, data pertaining to bone fractures, especially among reproductive-age women in Malaysia, are very limited. Micronutrients like calcium, magnesium and phosphorus play vital roles in bone health, influencing bone mineral density and fracture risk. The objective of this study was to determine the prevalence of bone fractures among reproductive-age women and the association with dietary micronutrient intakes.

*Methods:* In this cross-sectional study, a total of 1,730 participants of reproductive-age women from the Malaysia Prospective Urban and Rural Epidemiological (PURE) study were recruited. The participants’ dietary intakes were assessed using a validated semi-quantitative food frequency questionnaire (FFQ). Selected micronutrients in the participants’ diets were calculated using the Malaysian food composition and the US Department of Agriculture food composition databases. The association between micronutrient intakes, comorbidities and physical activity levels with bone fractures were evaluated to identify predictors of bone fractures among reproductive-age women.

*Results:* The prevalence of bone fractures among Malaysian reproductive-age women was low (3.7%). The multiple logistic regression analysis showed that none of the micronutrients was associated with bone fractures. However, factors of diabetes and passive smoking in this study showed 2.6- and 4.0-times-higher odds of having bone fractures, respectively (AOR 2.580; 95% CI: 1.173–5.672) and (AOR 4.012; 95% CI: 2.265–7.107).

*Conclusions:* It was found that the majority of women in this study were taking lower micronutrient intakes of calcium, magnesium, and vitamin K than the Malaysia recommended nutrient intakes (RNI). Although this study showed that a low micronutrient intake is not significantly associated with bone fractures, it is recommended that future studies focus on controlled trials or prospective data analyses to establish causal relationships and the optimal micronutrient requirements for maintaining strong and healthy bones in women of reproductive age.

## Introduction

Bone fractures represent a significant health issue impacting individuals’ well-being and imposing a considerable burden on healthcare systems globally. Bone fracture is a medical condition in which there is a partial or complete break in the continuity of the bone [1]. In 2019, the global incidence of new fractures reached 178 million cases, reflecting a substantial increase of 33.4% since 1990. Furthermore, it has been reported that the prevalence of bone fractures had risen to 455 million cases by 2019, indicating a significant (70%) increase since 1990 [1].

While fractures in both children and older adults have received significant attention in the research, there has been relatively little focus on the population in between, specifically reproductive-age individuals. Reproductive-age women are those women aged between 15 and 49 years old, according to the World Health Organization. This age group, which encompasses individuals in their late teens to early forties, is a critical stage in terms of bone health [2]. A study conducted among reproductive-age women (mean age 21.34 ± 0.83 years old) in Saudi Arabia showed that 33% of the studied population had been diagnosed with either osteopenia or osteoporosis. The significant occurrence of osteopenia in reproductive-age women highlights the immediate requirement for early intervention to prevent the onset of osteoporosis in the future [3].

However, data pertaining to bone fractures, especially among reproductive-age women in Malaysia, are very limited. A study conducted in Klang Valley among adult women age 40 years old and older, with the mean age of 57.16 (SD = 9.12) years old, showed that the prevalence of osteoporosis was 16.1%, which is lower compared to the results of the study in Saudi Arabia [4]. During their reproductive age, women will undergo important physiological changes and engage in various activities that can influence their bone health and fracture risk: Factors such as hormonal fluctuations, lifestyle choices and nutrient deficiencies can all impact bone density and fracture susceptibility.

Micronutrients are the essential nutrients required by the body in small amounts for various physiological functions. They also play a crucial role in maintaining optimal bone health. Although these essential nutrients are required in small quantities, they have significant impacts on bone development, maintenance and overall strength. Several micronutrients, namely, calcium, magnesium, phosphorus, vitamin K and vitamin C, have been associated with various aspects of bone health, including bone mineral density, bone formation and decreased fracture risk [5].

A comprehensive pooled analysis study involving data from 22 countries revealed that approximately 69% of non-pregnant women in their reproductive age were estimated to have deficiencies in at least one of the three essential micronutrients, which are iron, zinc and vitamin A. This estimation indicates a staggering number of approximately 1.2 billion (with a range of 1.0–1.4 billion) non-pregnant women in reproductive age who are affected by micronutrient deficiencies [6]. This shows that micronutrient deficiency is one of the major health issues of reproductive age.

By focusing on the relationship between micronutrients and the risk of bone fractures, the objectives of this study were to determine the prevalence of bone fractures among reproductive-age women and to investigate its association with dietary micronutrient intakes of calcium, magnesium, phosphorus, vitamin K and vitamin C.

## Methodology

The description of the PURE study has been described elsewhere [7, 8]. This study is an extensive international research project that aims to examine the occurrence, death rates and risk factors related to non-communicable diseases. It involves participants from both urban and rural communities in 27 countries, including Malaysia. The Population Health Research Institute (PHRI) in Hamilton, Ontario, Canada, serves as the coordinating centre for the PURE study. In Malaysia, two universities have collaborated in handling data collection; they are Universiti Kebangsaan Malaysia (UKM) and Universiti Teknologi MARA (UiTM). Since 2007, a total of 15,792 Malaysian citizens, aged between 35 and 70 years old, had been enrolled in the study, and follow-up data collection will be continued until 2030.

Participants were recruited from selected urban and rural areas across both peninsular and east Malaysia. Following permission from community leaders, health screening and promotion booths were established in the assembly halls of these communities. With the assistance of community leaders, residents were notified and encouraged to visit the booths. Interested individuals who met the eligibility criteria were given a briefing about the study. Once written informed consent was obtained, participants underwent medical history assessments and basic physical examinations, and home visits were scheduled. During these home visits, other individuals residing in the household were invited to join the study. To ensure the feasibility of long-term follow-up, only household members intending to continue residing in their current homes for an additional 4 years were selected to participate. All participants provided written informed consent after they comprehended that their involvement was entirely voluntary [8].

**Figure 1:**
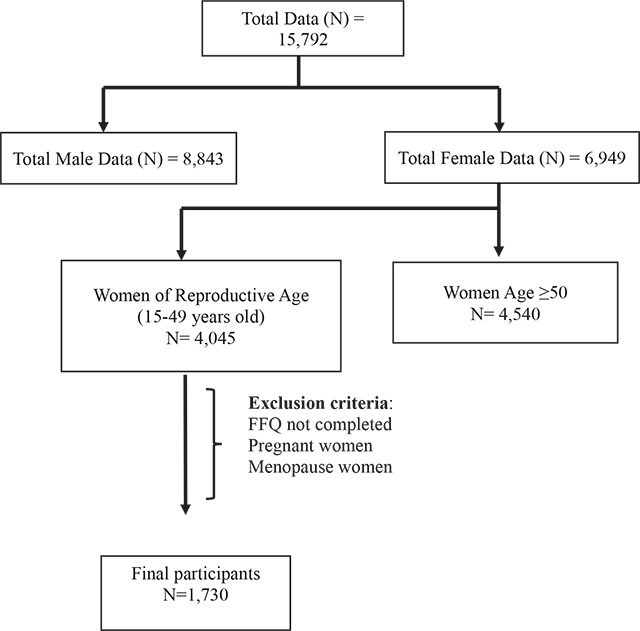
Flow chart of sample selection.

In this study, the focus was on reproductive-age women. Based on the World Health Organization’s definition, reproductive-age women are women from 15 to 49 years old [9]. However, in the PURE study, the recruited participants were adults aged between 35 and 70 years old; therefore only participants aged between 35 and 49 were included in this study. A total of 1,730 respondents were recruited, as shown in Figure 1. In order to maintain consistent and standardized data-collection procedures, research assistants underwent comprehensive training using operation manuals, videos and workshops. The collected data were then electronically transferred to the project office and to the coordinating center at the Population Health Research Institute (PHRI) in Hamilton, Ontario, Canada, for quality-control purposes. The research protocol received approval from the Hamilton Health Sciences Research Ethics Board (PHRI), the Research and Ethics Committee at UKM Medical Centre and the Research Ethics Committee at UiTM (Project code: PHUM-2012-01).

## Measurements

Participants were defined as having a bone fracture if they answered yes to the question “Have you ever fractured a bone?.” The questionnaire used in this study was developed by the Population Health Research Institute (PHRI) and subsequently revised and validated by the Malaysian team of researchers to ensure its appropriateness for the local population. Facial and content-validity assessments were conducted by the research team, which consisted of experts in public health–related studies. The validated PURE questionnaire was employed to gather information on demographics, personal medical history, and lifestyle factors (smoking status: active or passive). Demographic characteristics included age, marital status (currently married or single or widowed), education level (lower or higher education), and employment status (employed or unemployed). The residency area (urban or rural) was determined based on local government-gazetted areas, with rural areas defined as regions with fewer than 150 residents per square kilometre.

To assess the dietary intake of participants, a validated semi-quantitative food frequency questionnaire (FFQ) was employed [10]. The participants were provided with a list of food and drink items and then asked to report the frequency and serving sizes of their consumption over the past year: “During the past year, on average, how often have you consumed the following foods or drinks?” Response options varied from “Never” to “More than 6 times/day.” To determine the nutrient content of the food and drinks consumed, we used both the Malaysian Food Composition (MyFCD) database and the US Department of Agriculture (USDA) food-composition database; these databases were adjusted as needed by incorporating data from local food-composition tables and nutrient databases, which included recipes for commonly consumed mixed dishes in the local area. The incorporation of this additional information was based on studies conducted by Miller et al. [10].

The validated International Physical Activity Questionnaire (IPAQ), as described in the studies by Yusuf and Islam [11] and by Dehghan and Mente [12], was used to assess the participants’ physical activity levels. Physical activity was categorized as low if it was below 600 Metabolic Equivalent (MET) minutes per week. Conversely, it was considered high if it equalled or exceeded 600 MET minutes per week [13].

To collect anthropometric data, height measurements were obtained using a portable stature meter, while weight measurements were taken using the TANITA (BC-558 Ironman^®^) segmental body composition analyzer. Both measurements were taken twice during the session to ensure accuracy, and the average value was recorded. Body mass index (BMI) was calculated by dividing weight in kilograms by height in meters squared. Non-obesity was defined as a BMI less than 30 kg/m^2^, and obesity was defined as a BMI equal to or greater than 30 kg/m^2^ [7].

## Statistical Analysis

The data analysis was performed using IBM SPSS Statistics version 27. Descriptive analysis was conducted to identify the general characteristics of the respondents. Categorical data were presented as frequencies and percentages, while continuous data were summarized using the median and the interquartile range. To assess the differences between the bone fracture group and the non–bone fracture group, the Pearson chi-squared test was employed for variables such as age, residency area, education level, employment status, marital status, BMI, physical activity, co-morbidity, smoking status (including active and passive smoking) and micronutrient variables that were categorized as below RNI or equal to and above RNI. The statistical significance level was set at *p* < 0.05. Subsequently, simple logistic regression analysis was used to determine the association between the factors and bone fracture status. Variables with a *p*-value <0.25 in simple logistic regression were included in the multiple logistic regression model, for which the prevalence of rejecting the null hypothesis was set at 0.05.

## Results

Table 1 shows that the prevalence of bone fractures among Malaysian reproductive-age women was 3.7% (*n* = 64). The median age of this study population was 43 (IQR [25th, 75th percentile]: 39, 46) years old. In general, a total of 91.4% of the women were married, and 79.6% of them had received an education up to secondary school. A total of 49.2% of the women were employed, and 54.7% of them lived in rural areas. A total of 5.9% and 14.3% of the women were diagnosed with diabetes mellitus and hypertension, respectively. Forty percent of the women were passive smokers, while 2.0% of them were active smokers. Meanwhile, 64.0% of the women had low physical activity levels, and 22.7% of them were categorised as obese.

**Table 1:** General characteristics of reproductive age women (*N* = 1,730).

CHARACTERISTICS	N (%)	MEDIAN (IQR: 25^TH^, 75^TH^ PERCENTILE)
**Sociodemography**		
**Age (year)**		43.00 (39, 46)
**Age category (year)**		
35–39	468 (27.1)	
40–44	652 (37.7)	
45–49	610 (35.2)	
**Location**		
Urban	783 (45.3)	
Rural	947 (54.7)	
**Education level**		
Low	1,377 (79.6)	
High	353 (20.4)	
**Employment status**^#^ **(*n* = 1,424)**		
Employed	701 (49.2)	
Unemployed	723 (50.8)	
**Marital status**		
Currently married	1,582 (91.4)	
Single and/or widowed	148 (8.6)	
**BMI**		
Obese	392 (22.7)	26.23 (22.88, 29.60)
Non-obese	1,338 (77.3)	
**Co-morbidity (diabetes mellitus)**		
Yes	102 (5.9)	
No	1,628 (94.1)	
**Co-morbidity (hypertension)**		
Yes	247 (14.3)	
No	1,483 (85.7)	
**Lifestyle**		
**Active smoking**^#^ **(*n* = 1,725)**		
Yes	35 (2.0)	
No	1,690 (98.0)	
**Passive smoking**^#^ **(*n* = 1,691)**		
Yes	678 (40.1)	
No	1,013 (59.9)	
**Physical activity**^#^ **(*n* = 1,628)**		
Low (<600 MET)	1,048 (64.4)	
High (≥600 MET)	580 (35.6)	
**Micronutrients**		
**Phosphorus**		
<RNI 700 mg/day	202 (11.7)	1,365.13 (913.62, 1929.78)
≥RNI 700 mg/day	1,528 (88.3)	
**Vitamin K**		
<RNI 55 μg/day	1,484 (85.8)	14.72 (3.76, 34.14)
≥RNI 55 μg/day	246 (14.2)	
**Calcium**		
<RNI 1,000 mg/day	1,471 (85.0)	578.80 (375.27, 805.89)
≥RNI 1,000 mg/day	259 (15.0)	
**Magnesium**		
<RNI 320 mg/day	1,725 (99.7)	28.21 (14.97, 53.38)
≥RNI 320 mg/day	5 (0.3)	
**Vitamin C**		
<RNI 70 mg/day	490 (27.3)	105.98 (62.43, 178.23)
≥RNI 70 mg/day	1,257 (72.7)	

*significant at *p*-value <0.05; #the total numbers (*n*) are not equal to 1,730 due to missing values

For the micronutrient consumption based on the recommended intakes (RNI), a total of 85.0%, 99.7% and 85.8% of the women, respectively, had calcium, magnesium, and vitamin K intakes lower than the RNI. On the other hand, 88.3% and 72.7% of the women, respectively, had phosphorus and vitamin C intakes higher than the RNI.

Table 2 shows that the micronutrient components of calcium, magnesium, phosphorus, vitamin K and vitamin C do not show a significant association with bone fractures. However, in this study, findings revealed that factors of diabetes and passive smoking were significantly associated with bone fractures, (*p* = 0.011) and (*p* < 0.001), respectively.

**Table 2:** Factors associated with bone fractures among reproductive-age women (*N* = 1,730).

CHARACTERISTICS	BONE FRACTURES, N (%)		
	YES	NO	*P*-VALUE
**Age category (year)**			
35–39	20 (4.3)	448 (95.7)	0.274
40–44	18 (2.8)	634 (97.2)	
45–49	26 (4.3)	584 (95.7)	

**Location**			
Urban	28 (3.6)	755 (96.4)	0.805
Rural	36 (3.8)	911 (96.2)	

**Education level**			
Low	46 (3.3)	1,521 (96.1)	0.118
High	18 (5.1)	145 (98.0)	

**Employment status**^#^ **(*n* = 1,424)**			
Employed	25 (3.6)	676 (96.4)	0.660
Unemployed	29 (4.0)	694 (96.0)	

**Marital status**			
Currently married	61(3.9)	1,521 (96.1)	0.260
Single and/or widowed	3 (2.0)	145 (98.0)	

**BMI**			
Obese	17 (4.3)	375 (95.7)	0.447
Non-obese	47 (3.5)	1,291 (96.5)	

**Co-morbidity (diabetes mellitus)**			
Yes	8 (7.8)	94 (92.2)	**0.044** ^a*^
No	56 (3.4)	1,572 (96.6)	

**Co-morbidity (hypertension)**			
Yes	10 (4.0)	237 (96.0)	0.754
No	54 (3.6)	1,429 (96.4)	
**Lifestyle**			
**Active smoking**^#^ **(*n* = 1,725)**			
Yes	1 (2.9)	34 (97.1)	1.000^a^
No	62 (3.7)	1,628 (96.3)	
**Passive smoking**^#^ **(*n* = 1,691)**			
Yes	43 (6.3)	635 (93.7)	<0.001*
No	17 (1.7)	996 (98.3)	
**Physical activity**^#^ **(*n* = 1,628)**			
Low	36 (3.4)	1,012 (96.6)	0.845
High	21 (3.6)	559 (96.4)	

**Micronutrients**			
**Minerals**			
**Calcium mg/day**			
<RNI 1,000 mg/day	55 (3.7)	1,416 (96.3)	0.836
≥RNI 1,000 mg/day	9 (3.5)	250 (96.5)	
**Trace elements**			
**Phosphorus mg/day**			
<RNI 700 mg/day	5 (2.5)	197 (97.5)	0.327
≥RNI 700 mg/day	59 (3.9)	1,469 (96.1)	
**Magnesium mg/day**			
<RNI 320 mg/day	64 (3.7)	1,661 (96.3)	1.000^a^
≥RNI 320 mg/day	0 (0)	5 (100.0)	
**Vitamins**			
**Vitamin C mg/day**			
<RNI 70 mg/day	23 (4.7)	467 (95.3)	0.168
≥RNI 70 mg/day	41 (3.3)	1,199 (96.7)	
**Vitamin K μg/day**			
<RNI 55 μg/day	58 (3.9)	1,426 (96.1)	0.258
≥RNI 55 μg/day	6 (2.4)	240 (97.6)	

*Significant at *p*-value <0.05; a = Yates correction; #the total numbers (*n*) are not equal to 1,730 due to missing values

## Factors Associated with Bone Fractures among Reproductive-Age Women

The simple logistic regression analysis showed that none of the micronutrient variables was significantly associated with bone fractures in the study population. Meanwhile, the analysis showed that significant factors associated with bone fractures among the population studied were women’s diabetic status (POR = 2.389, 95% CI: 1.107–5.157) and passive smoking status (POR = 3.967, 95% CI: 2.243–7.017) (Table 3).

**Table 3:** Predictors of bone fractures among reproductive-age women.

CHARACTERISTICS	BONE FRACTURES, *N* = 64 (3.7%)					
	SIMPLE LOGISTIC REGRESSION			MULTIPLE LOGISTIC REGRESSION		
	CRUDE OR 95% CI	WALD (DF)	*P*-VALUE	ADJ OR 95% CI	WALD (DF)	*P*-VALUE
**Age category (year)**						
35–39	1					
40–44	0.636 (0.333, 1.216)	1.873 (1)	0.171			
45–49	0.997 (0.550, 1.810)	0.000 (1)	0.993			
**Residency**						
Urban	0.938 (0.567, 1.552)	0.061 (1)	0.805			
Rural	1					
**Education level**						
Low	1					
High	1.555 (–0.890, 2.716)	2.403 (1)	0.121			
**Employment status** ^#^						
Employed	1					
Unemployed	0.885 (0.513, 1.527)	0.193 (1)	0.661			
**Marital status** ^#^						
Currently married	1.938 (0.601, 6.255)	1.226 (1)	0.268			
Single and/or widowed	1					
**BMI**						
Obese	1.245 (0.707, 2.194)	0.576 (1)	0.448			
Non-obese	1					
**Co-morbidity (diabetes mellitus)**						
Yes	2.389 (1.107, 5.157)	4.921 (1)	0.027*	2.580 (1.173, 5.672)	5.559 (1)	0.018*
No	1			1		
**Co-morbidity (hypertension)** ^#^						
Yes	1.117 (0.561, 2.223)	0.099 (1)	0.754			
No	1					
**Lifestyle Active smoking** ^#^						
Yes	0.772 (0.104, 5.733)	0.064 (1)	0.801			
No	1					
**Passive smoking** ^#^						
Yes	3.967 (2.243, 7.017)	22.434 (1)	<0.001*	4.012 (2.265, 7.107)	22.673 (1)	<0.001*
No	1			1		
**Physical activity**						
Low	1.056 (0.611, 1.827)	0.038 (1)	0.845			
High	1					

**Micronutrients**						
**Minerals**						
Calcium mg/day						
<RNI 1,000 mg/day	1.079 (0.527, 2.211)	0.043 (1)	0.836			
≥RNI 1,000 mg/day	1					

**Trace elements**						
**Phosphorus mg/day**						
<RNI 700 mg/day	0.632 (0.251, 1.594)	0.946 (1)	0.331			
≥RNI 700 mg/day	1					
**Magnesium mg/day**						
<RNI 320 mg/day	62245871.77 (0.000)	0.000 (1)	0.999			
≥RNI 320 mg/day	1					
**Vitamins**						
**Vitamin C** **mg/day**						
<RNI 70 mg/day	1.440 (0.855, 2.427)	1.879 (1)	0.170			
≥RNI 70 mg/day	1					

**Vitamin K μg/day**						
<RNI 55 μg/day	1.627 (0.694, 3.812)	1.255 (1)	0.263			
≥RNI 55 μg/day	1					

*Significant at *p*-value <0.05

The study proceeds with multiple logistic regression analyses to come up with a predictive model for the risk of bone fractures in Malaysian reproductive-age women (Table 3). This multivariate analysis assessed multiple variable associations with bone fractures after adjusting for confounders to obtain an adjusted odds ratio (AOR). In the simple logistic regression, all variables that were significant (*p* < 0.05) and those with a *p*-value <0.25 were included in the multiple logistic regression analysis. The variables were age, education level, being diagnosed with diabetes mellitus, being a passive smoker and micronutrient intakes of vitamin C.

The multiple logistic regression analysis showed that none of the micronutrient intakes was included in the final model, but only two other factors were included in the final model: diabetic women, who had nearly 2.6-times-higher odds of having bone fractures than non-diabetic women (AOR 2.580; 95% CI: 1.173–5.672) and women who were passive smokers, who had four-times-higher odds of having bone fractures compared to women who were not exposed to tobacco smoke (AOR 4.012; 95% CI: 2.265–7.107). There was no interaction between the variables.

## Discussion

The prevalence of bone fractures among Malaysian reproductive-age women (35–49 years old) in this study was 3.7%. However, no studies related to bone fractures among the reproductive-age women have been found in Malaysia for comparison. Nevertheless, a cross-sectional study conducted in China by Wang and Yu [14] showed that the prevalence of fractures among women aged between 40 and 49 years old was 4.3%, and the overall prevalence of bone fractures in the study for individuals above 40 years old was 20.6% (95% CI: 19.3–22). This study provides additional insight into fracture rates among women of similar age groups. Another study conducted by Waterloo and Ahmed [15] examined the prevalence of bone fractures, including among reproductive-age women and reported a prevalence of 3.4% for women below 60 years old. The findings from this study are aligned with the current study, indicating a similar trend.

### Micronutrients

Micronutrient intakes play a crucial role in bone health and can have a significant impact on the risk of bone fractures in reproductive-age women. The five most important micronutrients related to bone health, namely, calcium, magnesium, phosphorus, vitamin K and vitamin C, are discussed.

### Calcium

Adequate calcium intake is essential for maintaining strong and healthy bones. Calcium is a key component of bone tissue, and a deficiency in calcium can lead to reduced bone density and an increased risk of fractures [16]. This study showed that 85.0% of the study population had calcium intakes below the RNI. The recommended calcium intake for women of reproductive age is 1,000 mg/day [17]. However, the median intake for this population was 578.80 mg/day, which is far below the recommended level.

A meta-analysis conducted to determine the global dietary calcium intake in adults showed that among the 74 countries included in the data, the average national intake of dietary calcium varies significantly, ranging from 175 mg/day to 1,233 mg/day. Several countries in Asia exhibit an average dietary calcium intake of less than 500 mg/day. On the other hand, countries in Africa and South America predominantly demonstrate lower calcium intake levels, typically ranging from approximately 400 mg/day to 700 mg/day [18]. This finding showed a similar trend with the present study, in which calcium intake is lower than the recommended level. Although the association between bone fractures and calcium intake is not significant in this present study, there is a trend of higher prevalence of bone fractures (3.7% vs. 3.5%) in the low-calcium-intake group.

A cross-sectional study conducted in Italy aimed to examine the relationship among calcium intake, bone mineral density (BMD), and fragility fractures and found no correlation between calcium intake and BMD. However, a significant association was found when comparing individuals with a daily dietary calcium intake below 400 mg/day to those with a daily intake of 400 mg/day or higher. The transition to a higher calcium intake was linked to a reduced fracture probability ratio at any site, with the risk decreasing from 42% to 21% (*p* < 0.05). Furthermore, individuals who had one or more vertebral fractures had a significantly lower dietary calcium intake (<400 mg/day) compared to those without vertebral fractures [19].

However, a systematic review study found that most studies examining the relationship between dietary calcium intake and fractures did not show a significant association. Out of the studies reviewed, 14 out of 22 reported no association with total fractures, in which 17 out of 21 studies reported no association with hip fractures; seven out of eight studies reported no association with vertebral fractures; and five out of seven studies reported no association with forearm fractures. However, when looking specifically at randomized controlled trials using calcium supplements, it was observed that these supplements reduced the risk of total fractures and vertebral fractures (20 studies, *n* = 58,573; relative risk 0.89, 95% CI: 0.81–0.96) and vertebral fractures (12 studies, *n* = 48 967; relative risk 0.86, 95% CI: 0.74–1.00) [20].

### Magnesium

Magnesium is involved in bone mineralization and thus plays a role in bone strength. It works synergistically with calcium and vitamin D to support optimal bone health. Insufficient magnesium intake may compromise bone density and increase the risk of fractures in reproductive-age women [21]. This present study reported that almost all the women (99.7%) in this study had magnesium intake below the RNI level [22]. The median intake for the study population was 28.21 mg/day, which is 11 times lower than the RNI (320 mg/day).

According to the study conducted by Beal and Massiot [23], Southeast Asia had the highest estimated prevalence of inadequate intake of calcium, folate, magnesium, riboflavin, and thiamine for all years between 1961 and 2011, a finding that corresponds with our present study’s finding. Our study showed that lower intake of magnesium did not significantly associate with bone fractures. However, other data showed that bone fractures tend to be higher with low magnesium intake compared to adequate intake (3.7% vs. 0%).

A cohort study investigating the link between dietary magnesium intake and fracture risk reported that women who met the recommended magnesium intake had a 27% lower risk of experiencing future fractures (95% CI: 0.51–0.98, *p* = 0.04). Furthermore, the study revealed that women with the highest magnesium intake had a significantly reduced risk of fractures, with a hazard ratio (HR) of 0.47 and a 95% CI: 0.33–0.68, *p* < 0.0001 [24].

In another cohort study investigating the association between magnesium levels and bone fractures, it was reported that low serum magnesium levels were linked to an elevated risk of fractures. Through Cox regression analysis, the HR for total fractures comparing the bottom quartile of magnesium concentrations to the top quartile was 2.10, with a 95% CI of 1.30–3.41. Based on these findings, the researchers concluded that low serum magnesium is significantly and independently associated with a higher risk of fractures [25].

### Phosphorus

Phosphorus plays a crucial role in bone health by aiding in the process of bone mineralization. It is a key component of hydroxyapatite, which is the mineralized matrix that gives bones their strength and rigidity. Alongside calcium, phosphorus provides structural support to bones and contributes to their overall health and integrity. Phosphorus is involved in maintaining the balance between bone formation and resorption, helping to regulate bone turnover and remodeling. Adequate levels of phosphorus are necessary for optimal bone mineralization, which is essential for maintaining strong and healthy bones throughout life [26].

The current study found that 88.3% of the reproductive-age women consumed phosphorus more or at the same amount as the RNI. The median phosphorus intake was 1,365.13 mg/day, which is nearly twice the RNI of 700 mg/day. The phosphorus intake in this study was higher when compared to the intake in another study done on adults among Universiti Malaysia Terengganu staff, which revealed that the study’s median intake was 904.6 mg/day [27]. The current study found no significant association between phosphorus intake and bone fractures, but bivariate analysis revealed a trend of lower bone fractures in intakes lower than the RNI (2.5% vs. 3.9%).

The association between high phosphorus levels and bone health was reported in a study that aimed to determine the independent connections between phosphorus intake and bone health indicators, including bone mineral content (BMC) and BMD. The study demonstrated that a higher phosphorus intake was linked to increased BMC among female teenagers (Q4 vs. Q1: BMC, 30.9 ± 1.1 vs. 29.0 ± 0.5 g, *p* = 0.001). Phosphorus intake was found to be positively associated with BMC and BMD, as well as with a reduced risk of osteoporosis in adults aged over 20 years [28].

### Vitamin K

Vitamin K is important for bone health as it assists in the production of proteins involved in bone mineralization. It helps activate osteocalcin, a protein required for proper calcium utilization in bone tissue. Low vitamin K intake may lead to impaired bone formation and an increased risk of fractures [29]. The data from this present study showed that 85.8% of the study population had vitamin K intake below the RNI. The median intake of vitamin K was 14.72 mcg/day, which is only 20% of the recommended value (55 mcg/day). This study showed that there was no significant association between vitamin K intake and bone fractures; however, it was observed that the prevalence of fractures was lower among women with vitamin K intakes higher than the RNI (2.4% vs. 3.9%).

A systematic review was conducted to explore the effect of vitamin K on bone health, with a particular emphasis on bone remodeling, mineral density, and fractures caused by fragility. Based on the study, the researchers concluded that in young and elderly women, inadequate vitamin K consumption appears to be linked to bone deterioration, indicating a potential beneficial effect of vitamin K on skeletal well-being [30].

A meta-analysis conducted in 2018 reported a significant inverse association between dietary vitamin K intake and the risk of fractures. The highest intake of vitamin K, when compared to the lowest intake, showed a relative risk (RR) of 0.78, with a 95% CI: 0.56–0.99. Additionally, the analysis examining the dose–response relationship indicated that for every 50 μg of increase in dietary vitamin K intake per day, the pooled RR of fractures was 0.97 (95% CI: 0.95 to 0.99). Therefore, the researcher concluded that higher dietary vitamin K intake may have a moderate effect in reducing the risk of fractures [31].

### Vitamin C

Vitamin C is a vital nutrient that plays multiple roles in promoting bone health. It is essential for the synthesis of collagen, a key protein that forms the structural framework of bones, contributing to their strength and flexibility. In addition to collagen formation, vitamin C supports bone formation by regulating the activity of cells involved in building new bone tissue [32]. Ensuring adequate vitamin C intake during reproductive age is essential for optimizing bone health in the long term. Building strong and healthy bones during this period can have long-lasting effects, reducing the risk of bone-related issues such as osteoporosis and fractures later in life.

In the current study, it was demonstrated that an adequate intake of vitamin C was observed in more than 72.0% of the population, with a median intake of 105.98 mg/day. This intake level exceeded the recommended intake for reproductive-age individuals in the RNI (70 mg/day) by 50%. In comparison, a population-based study utilizing data from the 2014 Malaysian Adult Nutrition Survey (MANS) reported that 28.1% of the respondents had consumed vitamin/mineral supplements [33]. It was observed that there was a higher prevalence of dietary supplement usage among women compared to men, with percentages of 32.1% and 24.3%, respectively, and vitamin C appeared as the most used vitamin/mineral supplement (15.7%), followed by multivitamin and multimineral supplements (8.9%). The elevated intake of vitamin C observed in the present study may be due to supplement intake.

The current study also showed a trend of higher prevalence of bone fractures among lower vitamin C intake; however, it was not statistically significant. Therefore, the association between inadequate vitamin C intake and a higher risk of bone fractures cannot be established. It was supported by a meta-analysis study conducted by Malmir and Shab-Bidar [34] that showed that dietary vitamin C intake in females was not significantly associated with hip fractures (overall RR = 0.90; 95% CI: 0.71–1.15, *p* = 0.394).

Meanwhile, a contradictory finding was reported in a meta-analysis conducted by Sun and Liu [35], whereby there was a significant association between dietary vitamin C intake and the lowered risk of hip fracture (Odd’s ratio [OR] = 0.73, 95% CI: 0.55–0.97, 12 = 69.1%). The analysis also revealed a linear dose–response relationship, indicating that for every 50 mg/day increase in vitamin C intake, there was a 5% statistically significant reduction in the risk of hip fracture (OR = 0.95, 95% CI: 0.91–1.00, *p* = 0.05).

### Diabetes mellitus

In the study population, 5.9% of the women were diabetic, and from the multivariate analysis, women who were diagnosed with diabetes had almost 2.6-times-higher odds of having bone fractures compared to non-diabetic women (AOR 2.580; 95% CI: 1.173–5.672). This result has a similar finding as a previous meta-analysis that reported that type 2 diabetes mellitus (T2DM) was related to an elevated risk of any fracture (summary relative risk = 1.05, 95% CI: 1.04–1.06) that increased with age, duration of diabetes, and insulin therapy [36]. Another significant finding of two large meta-analyses involving 1.3 million people reported that T2DM patients have a moderately elevated risk of hip fractures (RR 1.7, 95% CI: 1.3–2.2; RR 1.38, 95% CI: 1.25–1.53, respectively) [37].

Despite having a normal or a higher body weight and enhanced bone mineral density, patients with T2DM are more prone to fragility fractures. The precise mechanisms underlying how T2DM affects bone fragility are still not completely known. The increased fractures seen in these women, however, are likely the result of several factors, including a higher risk of falls, a regional decline in bone density, and deteriorated bone quality. Additionally, the risk of fractures may be impacted by the medications used to treat T2DM [38]. To create efficient fracture-prevention measures for people with T2DM, more study is required to fully understand these interactions.

### Smoking

The involuntary inhaling of smoke from cigarettes or other tobacco products smoked by others is referred to as passive smoking. This study showed that 40.0% of women in this population had been exposed to smoke by the people surrounding them. The results from the multivariate analysis showed that passive smokers had a four-times-higher risk of bone fractures compared to those who were not exposed to tobacco smoke (AOR 4.012; 95% CI: 2.265–7.107).

Reduced BMD and the deterioration of bone tissue can lead to progressive fragility and an increased risk of fractures, which have been linked to the effect of smoking, which is recognized as a significant contributing risk factor. Smoking disrupts the delicate balance between bone resorption and formation, resulting in inadequate replacement of the resorbed bone, ultimately leading to low BMD. While limited research exists on the pathophysiological mechanisms through which smoking induces bone loss and on the clinical correlation between them, it is believed that smoking affects bone density through various pathways [39].

A cross-sectional study conducted in Korea to investigate the association between passive smoking and BMD discovered that T-scores across the entire femur (*p* = 0.001), femoral neck (*p* = 0.001) and lumbar spine (*p* = 0.004) were lower in passive smokers than in non-exposed groups. Impaired bone health (osteopenia or osteoporosis) was higher in passive smokers compared to non-exposed individuals (*p* = 0.004) (61.7% vs. 48.6%) [40].

A cohort study conducted over a period of 28 years among individuals from ages 3 to 18, at baseline from 31 to 46 years old, also reported the impact of passive hand smoking. The study revealed that individuals exposed to parental smoking had an increased risk of experiencing low-energy fractures, with an OR of 1.28 and a 95% CI ranging from 1.01 to 1.62. Additionally, childhood exposure to parental smoking was linked to a lower heel ultrasound-estimated bone mineral density in adulthood. The regression analysis indicated a negative impact, with a beta coefficient (β) of –0.097 ± 0.041 per smoking parent. This association was statistically significant (*p* = 0.02) [41].

This study has several limitations, however. First, the cross-sectional design does not allow for the determination of causality between micronutrient intake and bone fractures and the lack of information regarding the mechanism of bone fractures, whether it is caused by high-impact or low-impact fractures that may influence the relationship with micronutrient intakes. Second, dietary intake was self-reported using a food frequency questionnaire (FFQ), which may be subject to recall bias and inaccuracies. Third, the study population was limited to women aged 35 to 49 years, which may not be representative of all reproductive-age women.

Additionally, the study did not account for potential confounding factors such as genetic predisposition and detailed lifestyle factors beyond smoking and physical activity. Future research should consider longitudinal designs and include a more comprehensive set of variables to better understand the relationship between micronutrient intake and bone health.

## Conclusion

The prevalence of bone fractures among Malaysian reproductive-age women in this study was low, which is 3.7%. It was found that the majority of women in this study were taking micronutrients (namely, calcium, magnesium and vitamin K) below the recommended nutrient intakes (RNI). Although this study showed that micronutrient intakes were not significantly associated with bone fractures, it is recommended that future studies focus on controlled trials or prospective data analyses to establish causal relationships and determine the optimal micronutrient requirements for maintaining strong and healthy bones in women of reproductive age. By emphasizing the importance of micronutrient intakes, healthcare professionals can help empower women to make informed dietary choices that support their long-term bone health and overall well-being.

## Figures and Tables

**Table:** 

PURE	Prospective Urban and Rural Epidemiological Study
RESTU	Risk Epidemiological Study
FFQs	Food Frequency Questionnaires
RNI	Recommended Nutrient Intakes
PHRI	Population Health Research Institute
MyFCD	Malaysian Food Composition Database
USDA	United States Department of Agriculture
IPAQ	International Physical Activity Questionnaire
MET	Metabolic Equivalent
BMI	Body Mass Index
T2DM	Type 2 Diabetes Mellitus
BMD	Bone Mineral Density
BMC	Bone Mineral Content
MANS	Malaysian Adult Nutrition Survey

## Data Availability

The data that support the findings of this study are available from PHRI, but restrictions apply to the availability of these data, which were used under license for the current study and, thus, are not publicly available. Data are, however, available from the corresponding author upon reasonable request and with permission from PHRI.
